# Symbiosis in Sustainable Agriculture: Can Olive Fruit Fly Bacterial Microbiome Be Useful in Pest Management?

**DOI:** 10.3390/microorganisms7080238

**Published:** 2019-08-03

**Authors:** Tânia Nobre

**Affiliations:** Laboratory of Entomology, Instituto de Ciências Agrárias e Ambientais Mediterrânicas, University of Évora, Apartado 94, 7002-554 Évora, Portugal; tnobre@uevora.pt; Tel.: +351-266-760-885

**Keywords:** animal-microorganism interactions, symbiotic, pest management, bacterial microbiome, olive pest, *Bactrocera oleae*

## Abstract

The applied importance of symbiosis has been gaining recognition. The relevance of symbiosis has been increasing in agriculture, in developing sustainable practices, including pest management. Insect symbiotic microorganisms’ taxonomical and functional diversity is high, and so is the potential of manipulation of these microbial partners in suppressing pest populations. These strategies, which rely on functional organisms inhabiting the insect, are intrinsically less susceptible to external environmental variations and hence likely to overcome some of the challenges posed by climate change. Rates of climate change in the Mediterranean Basin are expected to exceed global trends for most variables, and this warming will also affect olive production and impact the interactions of olives and their main pest, the obligate olive fruit fly (*Bactrocera* *oleae*). This work summarizes the current knowledge on olive fly symbiotic bacteria towards the potential development of symbiosis-based strategies for olive fruit fly control. Particular emphasis is given to *Candidatus* Erwinia dacicola, an obligate, vertically transmitted endosymbiont that allows the insect to cope with the olive-plant produced defensive compound oleuropein, as a most promising target for a symbiosis disruption approach.

## 1. Introduction

The notion of biological-individual is crucial to all fields of life sciences. Each individual is better seen in the frame of interactive relationships among species, and the individual boundaries are shifting to accommodate a symbiotic view of the organism and of life itself (e.g., [1,2,3]). Symbiosis was first used in 1879 by Anton de Bary [4] to describe close, long-term associations between different organisms living together. The definition of symbiosis, however, has since then always faced controversy (e.g., [5,6]). In the present article, I refer to symbiosis broadly (independently if the relation is in a mutualistic or antagonistic manner) as a way to highlight that symbionts cannot be classified exclusively as beneficial, harmless or deleterious (reviewed in [7]), because impact on their partners can be affected by context (e.g., [8,9,10]).

The applied importance of symbiosis has been gaining recognition over time, becoming more relevant in regard to sustainable agriculture practices that can respond to modern challenges. Human population growth and climate change are amongst the most discussed challenges that global agriculture must face, and efficiency and resilience of crop production needs to be improved. At the same time, there is also an increasing awareness, from the consumer side, on the linkages between food demand and dietary choice, global food security, and environmental outcomes (including fertilizer and pesticide inputs, water use and climate change (e.g., [11,12,13,14])).

Enhancing plant productivity and resilience by stimulating the presence of beneficial soil biota has been a focus of interest, and the alteration of agricultural practices such as crop-rotation and tillage intensity has been proposed as a way to select particular groups of microorganisms (e.g., [15,16]; but see [17,18] for ongoing discussion). A great majority of the research focuses on plant–mycorrhizal fungus interactions, as they are long known to affect multiple functions and processes, as well as on legume–rhizobia symbiosis for the fixation of atmospheric nitrogen (N_2_). Other endophytes are also raising more attention, however (e.g., [19,20,21]). In addition to in-field practices to promote functional symbiosis, breeding for symbiosis is also beginning to gain relevance (e.g., [22,23]). The necessary sustainable increase in production will have to occur while climate is changing and becoming less predictable.

Overall rising temperatures, warmer winter minimum temperatures, changes in precipitation patterns, and water shortages [24] will affect aggressiveness and spreading of insect pests in largely unknown ways and highly species specific [25]. Changes in climate variability may also be significant, affecting the predictability and amplitude of outbreaks, but also the activity and abundance of natural enemies (e.g., [26,27]). Together, this may affect the efficacy of current crop protection technologies, and pest control measures such as host–plant resistance, natural enemies, bio-pesticides and synthetic chemicals are likely to change as a result of climate change. Strategies relying on the functional organisms that inhabit potential pest insects are intrinsically less susceptible to external environmental variations. However, their study is still in its infancy, and additional research is needed to incorporate such strategies into agricultural practices.

## 2. Symbiotic Microorganisms in Pest Control

Insects host a wide variety of microorganisms, resulting in many transient and some persistent relationships: insects display the full scope of endosymbiosis (see [28] for a review). The symbionts living in an insect’s internal environmental (intracellular, even in gut environments) have their conditions and resources largely controlled by the host [29,30,31] as the eukaryotic host provides for a (more) stable and isolated environment [32] (even though physico-chemical conditions influenced by different gut compartments can display extreme gradients of oxygen, hydrogen and pH; e.g., [33,34,35,36,37,38]). Particularly among symbionts inhabiting gut environments, this does not mean that they inhabit a simplified world; on the contrary, most insects molt during larval stages (holometaboulos, comprising nearly 85% of insect diversity [39]; but there are also hemimetabolous species, that molt at nymphal stages and a small number of ametabolous species where molting occurs throughout life). During molting, the peeling of the exoskeletal lining of the gut occurs, thus disturbing any attached bacterial populations.

The gut microbiota of insects is taxonomically diverse: not only are bacteria associated with the gut lumen but also protists, fungi and archaea (these are mainly associated with termites or other insects that feed on wood or detritus; e.g., [40,41]). Nonetheless, the great majority of the symbionts in insect guts are bacteria, and they vary immensely in total size, composition, locations and functions within the gut (reviewed in [42]).). Insect symbionts are classically seen as entangled in nutritional relationships (e.g., [42,43,44]), aiding in defence by boosting host immune systems and enhancing pathogens and parasitoid resistance (e.g., [45,46,47]), as well as in resistance to chemicals (e.g., [48,49]). Symbiotic microorganisms have also been shown to interfere in courtship and reproduction (e.g., [50,51,52]), may have a role in supplementing oxygen [53] and in governing host thermal tolerance [54]. Whatever the function of the symbiont, if the relationship is required for host survival or increased fitness, alterations on the symbiont will have an impact on the insect. In the particular case of insects with pest status, the manipulation of selected symbionts can be a tool for population control. Arora and Douglas [55] recently provided a critical review of multiple approaches available for insect pest control founded on manipulation of microbial partners. For diversity simplification, they clustered the associations between insects and microorganisms by transmission mode (horizontal vs vertical) and location (gut lumen vs internal tissues) (this classification refers only to endosymbionts, insect–microorganism symbiosis where symbionts are not within the body or cells of the host (e.g., [56,57])—ectosymbiosis is not considered).

In addition to transmission mode and location, the degree of host–symbiont dependence is crucial to the design of insect management strategies. Endosymbionts can either be obligate or facultative (reviewed in [58]). Obligate endosymbionts are those where both partners fully depend on each other—the symbiont cannot be found outside the host, and the host, if depleted of its symbiont, suffers severe consequences for survival and/or reproduction. Obligate endosymbionts usually live in specialized host cells (bacteriocytes), and the evolution of the relationship involves specialization on the part of both host and symbiont [59]. In these cases, the transmission of the bacteriocyte endosymbionts occurs vertically, and eggs or young embryos are infected by the bacteria [60]. This mode of transmission, associated with dependence level, is particularly interesting for developing strategies that disrupt this relationship. Once deprived of its symbionts, an insect cannot find an alternative source. Finding routes to disrupt symbiosis requires, per insect species, the identification of specific targets, and the screening for symbiosis-active compounds [61].

Although this maybe the most obvious strategy, the disturbance of the obligate microbial symbiotic partner might not always be suitable. Other strategies may include genetic modification of the symbiont or construction of heterologous associations (i.e., association with microbial partners that naturally would not occur). A comprehensive review of these three strategies, towards their potential in suppressing the insect pest population or in changing specific traits of the insect pests, can be found in [55].

As the challenges in pest control intensify, powered by the evolutionary arms race [62] between humans and pests involving pesticide resistance, and the increasing denial of pesticides’ environmental side-effects, we need to search for new approaches in insect pest management. Specificity and effectiveness are keywords in any strategy, and while specificity needs to be present in any approach, effectiveness can result from a strategy targeting synergies between approaches. This work intends to stimulate debate and encourage reconsideration of the potentialities of using symbionts in olive fruit fly management.

## 3. The Iconic Mediterranean Crop Olives and Its Main Insect Pest

World olive growing is estimated of around 1000 million olive trees, occupying an area of 10.2 million hectares with more than 90% of the total area is located in the Mediterranean Basin [63]. Edible olives and the production of olive oil dates back to 5000 BC [64,65]. For the Mediterranean region, three main olive pests have been recognized: The olive fruit fly, *Bactrocera oleae* (Rossi, 1790), the olive moth, *Prays oleae* (Bernard, 1788) and the black scale, *Saissetia oleae* (Olivier, 1791) [66,67,68]. The importance of the last two has decreased as a whole due to advances in olive pest management [67], although regional relevance persists [69,70,71]. The olive fruit fly, specialized to become monophagous, remains the most important olive tree pest. Production losses are estimated on an average of more than 15% yearly [72], and this fly has been responsible for losses of up to 80% of oil value and 100% of some table cultivars [73]. The impact of the olive fruit fly on olive products goes beyond direct production losses, as they can affect their quality, composition and inherent properties, also reverting in indirect losses to the producers (reviewed in [74]). Conventional olive fruit fly management strategies include the use of baits, attracting the olive fruit fly by colours and/or pheromones, but mainly the use of insecticides, and particularly over the last decades through the use of organophosphates (OPs) [67,73]. Resistance to the most commonly used OP, dimethoate, had evolved involving mutations of the *Ace* gene, which codes for acetylcholinesterase, the target enzyme of OPs and other insecticides (e.g., [75,76,77,78]). Olive fruit fly resistance to other types of insecticides, including pyrethroids (i.e., alpha-cypermethrin), has also been encountered [79], as well as to spinosad, a relatively new insecticide derived from the actinobacterium *Saccharopolyspora spinosa* [80]. It is thus clear that the olive fruit fly is capable of fast developing resistance to commonly used insecticides.

Rates of climate change in the Mediterranean Basin are expected to exceed global trends for most variables [24], and this change will certainly also affect olive production. Irrigation requirements will increase [81] as well as the risk of heat stress around flowering and the lack of cold weather that is required for blossoming [82]. Climate change will also impact the interactions of the olive and its pests, including the obligate olive fruit fly. Worsening the infestation problem, the expected increases in fly infestation levels in some areas [83] are likely to drive increased insecticide use and the consequent development/spreading of resistance. Therefore, the development of non-insecticidal pest management methods is crucial for the reduction of selection pressures on already-resistant populations (limiting the generalization of resistance), to avoid the development of new resistance mechanisms and to face the challenges that come with infestation dynamics due to climate change and associated costs and losses.

Non-insecticidal alternatives that have worked in some situations or shown potential include (a) mass trapping programs [84,85,86], (b) sterile insect technique (SIT) [87,88,89], (c) particle film [90,91,92] and (d) biological control using natural enemies [66]. Symbiosis-based strategies are still under developed, albeit attempts with the use of copper products as symbionticides [92,93,94]. Additionally to a disturbance of the *Ca.* Erwinia dacicola community, Bigiotti and co-workers [93] also reported that copper acted negatively on fly physiology, including egg production activity. However, copper is a commonly used fungicide in olive groves for more than a century, applied more than once in a growing season [95,96] which raises the question on its field capacity to control the olive fruit fly. Copper is non-specific and has several side-effects, and its long-term application resulted in the contamination of agricultural soils presenting nowadays a major environmental and toxicological concern (e.g., [96,97]).

## 4. Symbiotic Bacteria and the Olive Fruit Fly

*Bactrocera oleae* is no exception, as to understand its evolution and behavior, it is also better seen as a group of genetically different entities. In particular, it evolved to harbor a vertically transmitted and obligate bacterial symbiont—*Candidatus* Erwinia dacicola [98] found in different populations of olive fruit fly from several countries [99,100,101]. *Ca.* Erwinia dacicola is believed to allow the insect to cope with abundant secondary metabolites, particularly the olive-plant-produced defensive compound oleuropein (a bitter and otherwise toxic phenolic glycoside) [102]. Oleuropein is at higher concentrations on unripe olives and decreases throughout the fruit maturation process. Ben-Yosef et al. [102] demonstrated that olive flies without *Ca.* Erwinia dacicola did not develop beyond the second instar on unripe fruits, but on ripe fruits were able to reach adulthood (albeit with reduced fitness). Furthermore, several genes, both from the host and from *Ca.* Erwinia dacicola, were found overexpressed in the olive fly larvae when feeding on unripe olives, including a number of genes encoding detoxification and digestive enzymes. In particular, *Ca.* Erwinia dacicola amino-acid metabolism seems to be activated while olive fly larvae are feeding on unripe olives [103]. The unique capacity of these flies to develop on unripe olives, together with the specificity of the symbiotic relation, suggests a vital contribution of this bacteria to the life cycle of this fly. Indeed *Ca.* Erwinia dacicola is present in all life stages of wild olive fruit flies, being thus maintained through natural changes in diet (see [99,104] for loss of symbiont if specimens reared on artificial diets) and host metamorphosis; it transitions between intracellular and extracellular lifestyles during specific stages of the host’s life cycle [99,105].

Olive fruit fly larvae are monophagous, feeding exclusively on the tissue of olive fruits, while adults are polyphagous generalists, feeding on various substrates such as nectar, honeydew, fruit and plant exudates, bacteria and even bird faeces (e.g., [106,107,108,109]). Olive fruit flies share diverse bacterial relationships with other fruit flies (Tephritidae, subfamilies Dacinae and Trypetinae), and traditional microbiological approaches have identified other bacteria of the genera *Lactobacillus*, *Micrococcus*, *Pseudomonas*, *Streptococcus*, *Citrobacter*, *Proteus*, *Providencia*, *Enterobacter*, *Hafnia*, *Klebsiella*, *Serratia*, *Pantoea* and *Xanthomonas* [110,111,112,113,114]. Putatively important in the olive fruit fly life cycle, their role requires confirmation and they are mostly considered facultative [115]. The diverse flora of symbiotic bacteria may supplement substitute food [116] and further aid in detoxification of secondary compounds or nitrogen fixation [102,117]. In the new metabarcoding era, more bacterial groups are being found associated with the olive fruit fly, but the character of some of the relations is yet to be established. Molecular analyses have established the consistent presence of *Acetobacter tropicalis* in Greek olive fruit fly adults [118], *Pseudomonas putida* and *Asaia* sp. in Italian olive fruit fly populations [101], *Enterobacter* sp. in United States olive fruit fly populations [105] and *Tatumella* sp. in olive fruit fly populations in the Mediterranean region [119]. These bacteria, other than the obligatory endosymbiont *Ca.* Erwinia dacicola, are likely to be acquired from the environment during feeding and they probably inhabit the gut.

Hence, the olive fly appears to have a specific, beneficial bacterial microbiome composed of the maternally vertical transmitted endosymbiont *Ca.* Erwinia dacicola and other bacteria, likely transmitted horizontally during feeding, that might be considered facultative endosymbionts and which may nonetheless perform a relevant role during host development. Similar to what is observed in other mainly vertically transmitted insect-microorganisms endosymbiosis (e.g., [120,121,122]), also in *Ca.* Erwinia dacicola events of horizontal transmission were recently described (in laboratory reared populations; [123,124]), which are of relevance in thinking olive fruit fly control on a symbiotic basis.

## 5. Symbiosis-Based Olive Fruit Fly Control

Targeting microbial partners of insect pests has as its goal the management of pests by eliminating the microorganisms required for sustained insect growth, reproduction and survival [55]. The most obvious target for this type of symbiosis-based approach to the olive fruit fly is *Ca.* Erwinia dacicola, an obligate symbiont allocated in a specific cephalic organ (oesophageal bulb or pharyngeal bulb) where it multiplies rapidly, forming masses that reach the midgut and are passed to the following generation at the oviposition [98,105,125]. The development of effectors that perturb the specific resident microbial partners and their interactions with the insect, called symbiocides [55], are particularly interesting for disruption of vertically transmitted symbiosis, as the treated insect host cannot acquire the symbiont horizontally from the environment. The way *Ca.* Erwinia dacicola is transmitted to the eggs during oviposition (bacterial cap-like mass is typically found around the egg’s micropile; [98]) may provide targets for disruption of this association. Since the first description of this symbiosis [125], we know that this transmission occurs after the eggs exit the oviduct through the terminal rectal tract; here, contractile perianal glands that are filled with bacteria release the bacterial masses onto the eggs’ surfaces which are ingested by the larvae during eclosion [98]. Disturbance of the *B. oleae*/*Ca.* Erwinia dacicola relation was already shown possible by means of copper [92,93,94] or propolis [93] but the lack of specificity and, in the specific case of copper, its contaminating effects on soil and water (see above), calls for the search of more targeted approaches. Recently, Konstantopolous and Cosmidis [126] proposed a consensus pharmacophore model for *Ca.* Erwinia dacicola designed for the identification of the possible common binding interactions in a series of potential targets towards symbiont mortality. This knowledge has the potential to deliver a highly targeted tool for disrupting the symbiosis.

Other routes may become available by means of genomics and transcriptomics through the advances that are being made in understanding this symbiosis [127,128,129]. Specific molecular targets are expected due to co-evolution of the olive fruit fly and *Ca.* Erwinia dacicola, and the forecast of these specificities is a strong motivation to develop molecular methods targeting this particular symbiosis.

Nonetheless, the other “less obvious” horizontal symbiotic microorganisms might also be considered in an attempt to design approaches to manage this insect pest. Most are transient microorganisms, but several have been repeatedly found in olive fruit fly populations (e.g., [101,118,119]), suggesting a somewhat obligate character of the interactions. These partners are of particular interest, as these horizontally transmissible gut microorganisms can potentially be used to deliver specific microbicidal agents against *Ca.* Erwinia dacicola.

Within the transient microorganisms of the olive fruit fly—some acquired in food and water, which then become located in the gut lumen—an extra window of opportunity might exist as heterologous associations (the experimental transfer of microorganisms from one insect species into another species that does not naturally harbor the microorganism) can be explored. Apostolaki et al. [130] reported for the first time the transinfection of olive fruit flies with a specific *Wolbachia* strain (known as an endosymbiont of the cherry fruit fly *Rhagoletis cerasi*). *Wolbachia* is well known by its capacity to manipulate host reproduction through a variety of processes (reviewed in [131]) and it is capable of inducing complete cytoplasmic incompatibility under laboratory conditions in the olive fruit fly host [130]. As the authors stressed, further research is needed, starting with upscaling population experiments to (1) assess *Wolbachia* transmission in mass rearing conditions, and (2) determine if reduction in the cage population is achieved. In practice, to be able to consider such an approach would require solid fundamental research on the incidence and mechanisms of microbial-mediated hybrid fitness and on the persistence and cross-transmission of the heterologous association. Mass rearing conditions of olive fruit fly, needed for these and other approaches, remain an extra challenge that requires optimization. Since the first success rearing reports in the 70’s (e.g., [132,133,134]), our understanding of the biology of the olive fruit fly has greatly increased. Optimizations to rearing protocols have been proposed [135,136,137] as the interest in renewed approaches for population management increases. Estes and co-workers [138] review and discuss the difficulties, improvements and future directions of *B. oleae* mass-rearing, and the role of the symbiotic microorganisms in successful rearing programs is now acknowledge (e.g., [135,139]).

If microbial partners of the olive fruit fly are found that are culturable, approaches based on the changes of insect traits by the genetic manipulation of associated microorganisms (paratransgenesis [140]) could also be considered [55]. Available genetic technologies have the potential to be applied to modify microorganisms in such a way that they will express specific traits virulent to the insect host; the potential of microorganisms to deliver dsRNA against essential genes of the insect pest has been demonstrated, as well as the exposure of the host to specific protein toxins produced by bacteria [141,142]. In practice, the development of these methods into an applicable tool is hindered by the potential for dissemination and the transmission of transformed microorganisms to other bacteria and into the environment. As such, approaches for risk reduction must come from a deeper understanding of genetic circuits and manipulation routes [55].

## 6. Conclusions

Symbiotic relationships between macro- and microorganisms are widespread in nature, and gaining acceptance and importance in applied fields. The potential of pest management through symbiotic microorganisms is promising, but requires deeper knowledge of the host insect, its microorganisms and the types and characteristics of the microorganism’s relationships.

The olive fruit fly is a relevant insect pest in the Mediterranean region, where current pest control strategies are failing. Alternative strategies based on symbiosis require consideration, with the obligate, vertical transmitted endosymbiont *Ca.* Erwinia dacicola being the most obvious candidate. The diversity and specificity of olive fruit fly interactions with symbiotic bacteria invites the search for a thorough understanding of the system, with the promise that these naturally evolved connections are ‘Les Liasons Dangereuses’, that we can search for a sustainable targeted pest management.
